# Fibrinogen: A Marker in Predicting Diabetic Foot Ulcer Severity

**DOI:** 10.1155/2016/2358321

**Published:** 2016-12-01

**Authors:** X. H. Li, L. Y. Guan, H. Y. Lin, S. H. Wang, Y. Q. Cao, X. Y. Jiang, Y. B. Wang

**Affiliations:** ^1^Health Management Center, Shandong Provincial Hospital Affiliated to Shandong University, Jinan, Shandong 250021, China; ^2^Department of Burn and Plastic Surgery, Shandong Provincial Hospital Affiliated to Shandong University, Jinan, Shandong 250021, China; ^3^Department of Endocrinology, Shandong Provincial Hospital Affiliated to Shandong University, Jinan, Shandong 250021, China

## Abstract

*Aims*. To examine whether fibrinogen levels are a valuable biomarker for assessing disease severity and monitoring disease progression in patients with diabetic foot ulcer (DFU).* Methods*. A retrospective study was designed to examine the utility of fibrinogen in estimating disease severity in patients with DFU admitted to our hospital between January 2015 and January 2016. In total, 152 patients with DFU were enrolled in the study group, and 52 age and gender matched people with diabetes but no DFU were included as the control group. DFU severity was assessed using Wagner criteria.* Results*. Patients with DFU were divided into 2 subgroups based on the Wagner criteria. Mean fibrinogen values were significantly higher in patients with DFU grade ≧ 3 compared to those with DFU grades 1-2 (5.23 ± 1.37 g/L versus 3.61 ± 1.04 g/L). Using ROC statistic, a cut-off value of 5.13 g/L indicated the possible amputation with a sensitivity of 81.8% and a specificity of 78.9% (positive predictive value [PPV] 78.6%, negative predictive value [89.0%]). Fibrinogen values were found to be correlated with CRP levels, neutrophil, and WBC count.* Conclusions*. Fibrinogen levels might be a valuable tool for assessing the disease severity and monitoring the disease progression in patients with DFU.

## 1. Introduction

Diabetic foot ulcer (DFU) is the complication of diabetes that impacts heavily on cost and quality of life of the people with diabetes. It is known that DFU remains the most common cause of nontraumatic lower extremity amputation [1–3]. It has been reported that the prevalence of DFU in the hospitalized patients ranging is from 4 to 10% and the risk of patients with diabetes developing a foot ulcer in their lifetime could be as high as 25% [4]. Early assessment of DFU in people with diabetes is crucial and still remains a difficult challenge for clinician.

In DFU, inflammatory events impair the wound healing process and polymorph nuclear neutrophils proliferation enhances tissue damage until chronic wounds develop. A DFU characteristic feature is an acute-phase reaction prolongation [5]. Acute-phase reactants C-reactive protein (CRP), white blood cell (WBC), neutrophil count, and platelet are commonly used as a predictor of amputation in the patients with DFU but have only modest accuracy in reflecting DFU disease severity [6, 7].

Another simple and effective marker of inflammation is acute-phase protein, fibrinogen, which has been estimated to be increased in patients with diabetic foot disease [8]. Although it has been demonstrated that patients with DFU have higher fibrinogen levels than those without ulcers [8, 9], more accurate investigation needs to be done to answer whether fibrinogen levels may be valuable to forecast the sequences in diabetic foot disease. The aim of this study was to examine the prognostic value of fibrinogen levels to monitor disease progression in the patients with DFU.

## 2. Methods

### 2.1. Study Participants

This retrospective study evaluated the diagnostic value of fibrinogen levels for disease severity in 152 DFU inpatients admitted to the Shandong Provincial Hospital affiliated to Shandong University from January 2015 to January 2016. The control group consisted of 52 people with diabetes who were admitted for diabetic complication rather than DFU, age and gender matched participants (male/female: 33/19). Although 206 patients with DFU were retrospectively reviewed, only 152 met study inclusion criteria, as they had a completed blood count with leukocyte differential and fibrinogen performed before initialing any treatment and also had medical records available. All 152 patients with DFU also fulfilled the following criteria: no prior treatment with corticosteroids; no hematological or neoplastic disorders.

DFU were graded according to Wagner's classification [10]. Grade 1: superficial ulcers not involving the tendon, capsule, or bone; grade 2: deep ulcers penetration to tendon or joint-capsule; grade 3: deep ulcers with abscess of osteomyelitis; grade 4: localized gangrene; grade 5: extensive gangrene requiring a major amputation.

### 2.2. Study Measurement

The data of age, sex, disease duration, and other medical history were extracted from hospital database. The physical examination comprised blood pressure (BP) and anthropometric measurements, including height, weight, and BMI. BMI was calculated as weight (kg) divided by height (m)^2^. The venous blood was drawn after 12 hours overnight fasting for examining of fasting plasma glucose (FPG) and glycated hemoglobin (HbA1c). Completed blood count, fibrinogen, and CRP were also recorded for each participant once admitted in hospital. Plasma fibrinogen was measured by immunoturbidimetric assay, and all the completed blood counting analysis was performed in the hematology laboratory of our hospital.

### 2.3. Statistical Analysis

Data analysis was performed by using SPSS 19.0 for Windows. The distribution of the different variables was examined for normality by the Kolmogorov-Smirnov test. Data are expressed as mean and standard error or percent. Variables with nonnormally distribution are expressed as geometric mean (95% confidence interval). All normally distributed data were analyzed using Student's* t*-test to evaluate differences in mean and chi-square test to evaluate differences in proportions. Data found to be nonnormally distributed were analyzed using Mann–Whitney* U* test for independent subgroups and the Wilcoxon test for dependent subgroups. Receiver operating characteristic (ROC) curve analysis was used to identify optimal cut-off values of fibrinogen, CRP, WBC, and neutrophil of amputation in the patients with DFU. Spearman's correlation analysis was done between fibrinogen and other inflammation markers. A *P* value of less than 0.05 was deemed statistically significant.

## 3. Results

The demographic and laboratory features of our DFU population (95 males and 57 females) and control group (33 males and 19 females) are summarized in Table 1. The mean age of the DFU and control groups was 65.7 ± 10.1 years and 62.0 ± 10.3 years, respectively. There were no statistically significant differences between the ages of the study participants. The mean fibrinogen values in patients with DFU and controls were 4.50 ± 1.49 g/L and 3.01 ± 1.07 g/L, respectively (*P* < 0.05). Inflammatory marker WBC and neutrophil were found to be significantly elevated in patients with DFU compared to controls. No significant differences were observed with respect to the levels of platelet between study group and control group.

**Figure 1:**
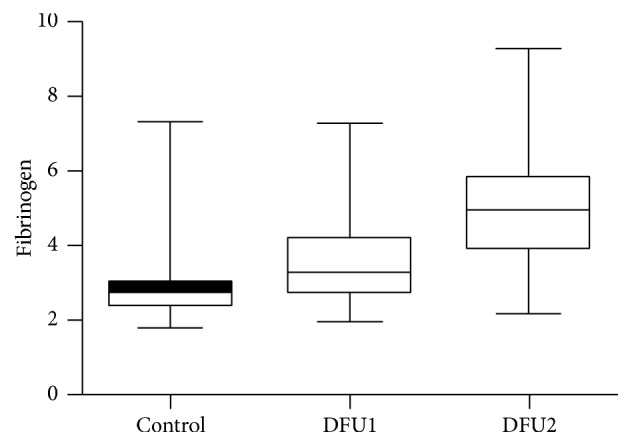
Box-plot representation of fibrinogen in patients with DFU grades 0-1 (DFU1) and DFU grade ≧ 2 (DFU2) and patients without DFU (control).

Table 2 shows mean fibrinogen values and the other inflammatory markers of study participants in the study. The total of 152 patients with DFU consisted of 80 with DFU grades 1-2 and 72 with DFU grade ≧ 3. In the group of DFU grade ≧ 3, fibrinogen values were found to be elevated compared to the group of DFU grades 1-2 and controls (5.23 ± 1.37 g/L, 3.61 ± 1.04 g/L, and 3.01 ± 1.07 g/L, resp.) (*P* < 0.05) (Figure 1). Significant differences were observed with respect to the levels of CRP, WBC, and neutrophil count between study participants.

Spearman correlation analysis indicated a significant correlation of fibrinogen with CRP (*r* = 0.705, *P* < 0.0001), neutrophil (*r* = 0.614, *P* < 0.0001), and WBC (*r* = 0.616, *P* < 0.0001) (Table 3). In patients with DFU grade ≧ 3, further analysis was also done between patients with CRP levels ≦ 10 mg/L and patient with CRP levels > 10 mg/L. A total of 21 patients with DFU grade ≧ 3 were found to have CRP levels ≦ 10 mg/L. Mean fibrinogen values of patients with DFU grade ≧ 3 and CRP levels > 10 mg/L (*n* = 59) were found to be higher (5.67 ± 1.48 g/L) than those with DFU grade ≧ 3 and CRP ≦ 10 mg/L (4.50 ± 1.24 g/L). Both of these levels were significantly higher than the group of patients with DFU grades 1-2 (3.49 ± 0.95 g/L) (*P* < 0.05).

**Figure 2:**
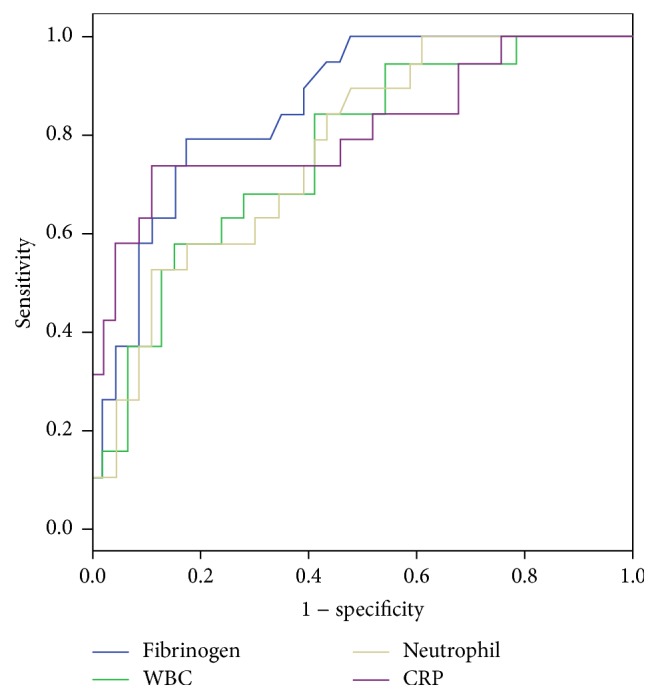
Receiver operating characteristic (ROC) curve of fibrinogen versus other inflammation markers in predicting amputation for patients with DFU.

During study period, 37 patients with DFU grade ≧ 3 and higher fibrinogen level (5.67 ± 1.31 g/L) had to undergo major or minor amputation due to poor wound healing. ROC curve analysis suggested that the optimum fibrinogen cut-off point for amputation in the total of 152 patients with DFU was 5.13 g/L, with sensitivity, specificity, PPV, and NPV of 80.9%, 82.6%, 78.6%, and 89.0%, respectively (AUC: 0.858) (Figure 2). The overall accuracy of fibrinogen in the determination of amputation was 83.6%. The same analysis for CRP, neutrophil, and WBC is summarized in Table 4.

## 4. Discussion

In this study, we evaluated fibrinogen as a marker of disease severity in patients with DFU. Our findings revealed that people with diabetes and DFU have elevated fibrinogen in comparison with people with diabetes but no DFU. An elevated level of fibrinogen was found to give high sensitivity, specificity, and predictive values in patients with DFU, which suggests a superiority of fibrinogen to CRP. CRP levels > 10 mg/L may reflect acute inflammation [11]. In our study, elevated fibrinogen values found in both groups of DFU grade ≧ 3 with and without elevated CRP levels prove that fibrinogen can be considered as an independent diagnostic marker for estimating disease severity, irrespective of CRP levels. The predictive superiority of fibrinogen that was found in our study can be attributed to its more stable nature compared to CRP.

Complications of foot ulcers are the major cause of hospitalization and amputation in the people with diabetes leading to significant health care costs as evidenced by the fact that 20–40% of health care resources are spent on diabetes-related diabetic foot [12]. In this study, the length of hospital stay for the patients undergoing amputation (22.5 ± 17.0 days) was significantly longer than that for the DFU patients without amputation (11.9 ± 8.8 days) (data not shown). It is therefore crucial to find effective markers for the assessment of disease severity and also for the tailoring of therapy. Although clinical, radiologic, and laboratory indices are used to assess disease severity in patients with patients, a great number of methods have also been investigated for DFU diagnosis and determination of disease severity [6]. Moreover, despite the role of inflammatory reaction in DFU pathogenesis, few data exist on the role of systemic inflammation in patients with DFU [13]. This study was designed to evaluate the role of fibrinogen, an acute-phase protein, in estimating DFU severity in conjunction with other clinical and biochemical severity indices.

Although there is no ideal single serum marker for predicting disease severity, WBC count, neutrophils count, platelet count, and CRP levels are used in routine clinical practice for determining DFU disease procession [3, 14]. These parameters can change according to the degree of the inflammation state, but they do not adequately reflect disease severity because of lacking data on their sensitivity and specificity. CRP seems to be more promising for predicting DFU severity and the outcome of treatment. A study by Lin et al. [15] studied 85 people with diabetes and a total of 90 infected limbs treated by percutaneous transluminal angioplasty (PTA). It has been found that limb salvage was successful in 66 cases while 24 underwent subsequent amputation. The study showed that the optimum cut-off CRP level in the major amputation group before PTA in patients with DFU was calculated to be 50 mg/L with a sensitivity and specificity of 70.7% and 81.8%, respectively, and concluded that the reduced CRP levels may serve as a major predictor of successful PTA outcome in people with diabetes and infected foot ulcers. More recent study reported that the cut-off CRP level in people with diabetes for the diagnosis of osteomyelitis is 14 mg/L with a sensitivity and specificity of 85% and 83% [3]. In the present study, the cut-off CRP level for estimating the amputation is 28.18 mg/L with overall accuracy of 80.1% (sensitivity 73.7%, specificity 89.1%).

Fibrinogen, an inflammatory marker, a major coagulation protein in the blood, and an important determinant of blood viscosity and platelet aggregation [16, 17], is a risk factor for vascular events [18]. Study by Kunutsor et al. [19] suggests that fibrinogen is positively, long-linearly, and independently associated with risk of sudden cardiac death. Apart from inflammatory processes, fibrinogen may also play a role in endothelial injury [20], the formation of low permeability fibrin clot [21], thrombosis [22], abnormalities of blood flow [23], and platelet hyperactivity [24] and contribute to the development of subclinical atherosclerosis [25]. Fibrinogen has recently been generally investigated in arterial coronary arterial disease and peripheral artery disease in people with diabetes [26, 27]. Data suggested that patients with diabetic foot disease have higher fibrinogen levels than those without ulcers [9]. Our data revealed a significant association between fibrinogen and DFU disease severity as reflected by amputation. Moreover, based on the finding of our study, we believe that a standardized cut-off value for fibrinogen in estimating DFU disease severity is crucial, as well as initial evaluation. Because of the need for starting an optimal treatment as soon as possible, elevated fibrinogen levels can give a significant clue to the clinician for estimating DFU disease severity.

Our study has several limitations. First, the present findings were based on analyses using a historical cohort; however, the patients were consecutively added to the cohort. Second, we did not evaluate time-dependent changes in plasma fibrinogen during the treatment period. Third, the number of the study subjects was relatively small. Fourth, this study was carried out in a single urban university hospital with limited representation, which may not be representative of the entire Chinese population with DFU.

In conclusion, the present study has demonstrated that fibrinogen levels are significantly elevated in patients with DFU and are correlated with clinical and laboratory indices. Fibrinogen in conjunction with other inflammatory markers may estimate DFU disease severity. If our data can be confirmed with further trials, we believe that a standardized cut-off value for fibrinogen would facilitate monitoring the disease progression. We therefore suggest that fibrinogen, as an inexpensive and easily applicable test, is a valuable tool for a rapid assessment of DFU disease severity to take timely and effective treatment.

## Figures and Tables

**Table 1:** Demographic and laboratory features of patients with patients and controls.

	DFU patients (*n* = 152)	Control group (*n* = 52)	*P* value
Age (years)	65.7 ± 10.1	62.0 ± 10.3	0.054
Gender (F/M)	57 (37.5%)/95 (62.5%)	19 (36.5%)/33 (63.5%)	0.902
Duration of diabetes (years)	12.8 (11.6, 14.1)	11.3 (8.8, 13.7)	0.249
Smoking (%)	37.40%	38.90%	0.781
Drinking (%)	35.00%	30.60%	0.7
BMI (kg/m^2^)	25.09 (24.28, 25.90)	25.21 (22.17, 28.25)	0.941
SBP (mmHg)	145.5 ± 22.7	140.8 ± 18.6	0.262
DBP (mmHg)	79.0 ± 15.8	83.7 ± 10.8	0.099
FBG (mmol/L)	7.6 (7.1, 8.1)	8.2 (7.2, 9.3)	0.277
HbA1c (%)	8.56 (8.18, 8.94)	8.54 (7.13, 9.95)	0.975
Hemoglobin (g/L)	123.4 ± 21.7	139.2 ± 18.3	<0.0001
WBC (×10^9^/L)	8.3 ± 3.8	6.7 ± 1.8	<0.0001
Neutrophil (×10^9^/L)	6.00 ± 3.61	3.79 ± 1.27	<0.0001
Platelet (×10^9^/L)	255.8 ± 79.5	233.5 ± 62.1	0.127
Fibrinogen (g/L)	4.50 ± 1.49	3.01 ± 1.07	<0.0001

Data were means ± SD or medians (interquartile range) or proportions for categorical variables. DFU: diabetic foot ulcer; SBP: systolic blood pressure; DBP: diastolic blood pressure; BMI: body mass index; FBG: fasting plasma glucose; HbA1c: glycosylated hemoglobin; WBC: white blood cell.

**Table 2:** Comparison of fibrinogen and other inflammation markers between patients with DFU (grades 1-2) and DFU (grade ≧ 3).

	DFU1 (*n* = 80)	DFU2 (*n* = 72)	*P* value
WBC (×10^9^/L)	6.95 ± 2.13	9.62 ± 4.31	<0.0001
Neutrophil (×10^9^/L)	4.66 ± 2.01	7.11 ± 4.23	<0.0001
CRP (mg/L)	9.01 ± 19.20	68.27 ± 85.82	<0.0001
Fibrinogen (g/L)	3.61 ± 1.04	5.23 ± 1.37	<0.0001

DFU1: diabetic foot ulcer (grades 1-2); DFU2: diabetic foot ulcer (grade ≧ 3); WBC: white blood cell; CRP: C-reactive protein.

**Table 3:** Spearman correlation coefficients between fibrinogen and other inflammation markers in patients with DFU.

	CRP	WBC	Neutrophil
Fibrinogen			
*r*_*s*_	0.705	0.616	0.614
*P*	<0.0001	<0.0001	<0.0001

WBC: white blood cells; CRP: C-reactive protein.

**Table 4:** Overall accuracy and ROC analyses of fibrinogen and other markers of inflammation to predict amputation from DFU.

	Cut-off	AUC	Sensitivity (%)	Specificity (%)	NPV (%)	PPV (%)	ACC (%)
Fibrinogen	5.13	0.858 (0.767, 0.950)	80.9	82.6	89.0	78.6	83.6
CRP	28.18	0.812 (0.684, 0.941)	73.7	89.1	87.9	67.6	80.1
WBC	9.87	0.765 (0.643, 0.888)	57.9	84.8	78.2	41.9	70.2
Neutrophil	7.86	0.771 (0.653, 0.888)	52.6	89.1	78.6	50.0	73.8

AUC: area under curve; ACC: accuracy; NPV: negative predictive value; PPV: positive predictive value; WBC: white blood cells; CRP: C-reactive protein.

## References

[1] Margolis D. J., Jeffcoate W. (2013). Epidemiology of foot ulceration and amputation: can global variation be explained?. *Medical Clinics of North America*.

[2] Martins-Mendes D., Monteiro-Soares M., Boyko E. J. (2014). The independent contribution of diabetic foot ulcer on lower extremity amputation and mortality risk. *Journal of Diabetes and Its Complications*.

[3] Michail M., Jude E., Liaskos C. (2013). The performance of serum inflammatory markers for the diagnosis and follow-up of patients with osteomyelitis. *International Journal of Lower Extremity Wounds*.

[4] Singh N., Armstrong D. G., Lipsky B. A. (2005). Preventing foot ulcers in patients with diabetes. *The Journal of the American Medical Association*.

[5] Mesa M. G., Duarte H. Á., Carretero J. H., Fors López M. M., Vilas M. M. (2011). De Marco Formula effectiveness as an adjunctive therapy to prevent infected ischemic diabetic foot amputation and reduce plasma fibrinogen. *Journal of Tissue Viability*.

[6] Demetriou M., Papanas N., Panopoulou M., Papatheodorou K., Maltezos E. (2013). Determinants of microbial load in infected diabetic foot ulcers: a pilot study. *International Journal of Endocrinology*.

[7] Tabur S., Eren M. A., Çelik Y. (2015). The major predictors of amputation and length of stay in diabetic patients with acute foot ulceration. *Wiener Klinische Wochenschrift*.

[8] Weigelt C., Rose B., Poschen U. (2009). Immune mediators in patients with acute diabetic foot syndrome. *Diabetes Care*.

[9] Rattan R., Nayak D. (2008). High levels of plasma malondialdehyde, protein carbonyl, and fibrinogen have prognostic potential to predict poor outcomes in patients with diabetic foot wounds: a preliminary communication. *International Journal of Lower Extremity Wounds*.

[10] Li X., Xiao T., Wang Y. (2011). Incidence, risk factors for amputation among patients with diabetic foot ulcer in a Chinese tertiary hospital. *Diabetes Research and Clinical Practice*.

[11] Pearson T. A., Mensah G. A., Alexander R. W. (2003). Markers of inflammation and cardiovascular disease: application to clinical and public health practice: a statement for healthcare professionals from the centers for disease control and prevention and the American Heart Association. *Circulation*.

[12] Lavery L. A., Armstrong D. G., Wunderlich R. P., Tredwell J., Boulton A. J. M. (2003). Diabetic foot syndrome: evaluating the prevalence and incidence of foot pathology in Mexican Americans and non-Hispanic whites from a diabetes disease management cohort. *Diabetes Care*.

[13] Tuttolomondo A., Maida C., Pinto A. (2015). Diabetic foot syndrome as a possible cardiovascular marker in diabetic patients. *Journal of Diabetes Research*.

[14] Wong K. L., Nather A., Liang S., Chang Z., Wong T. T. C., Lim C. T. (2013). Clinical outcomes of below knee amputations in diabetic foot patients. *Annals of the Academy of Medicine Singapore*.

[15] Lin C.-W., Hsu L.-A., Chen C.-C. (2010). C-reactive protein as an outcome predictor for percutaneous transluminal angioplasty in diabetic patients with peripheral arterial disease and infected foot ulcers. *Diabetes Research and Clinical Practice*.

[16] Smith E. B. (1995). Fibrinogen, fibrin and the arterial wall. *European Heart Journal*.

[17] Lowe G. D. O. (1995). Fibrinogen and cardiovascular disease: historical introduction. *European Heart Journal*.

[18] Danesh J., Lewington S., Thompson S. G. (2005). Plasma fibrinogen level and the risk of major cardiovascular diseases and nonvascular mortality: an individual participant meta-analysis. *JAMA*.

[19] Kunutsor S. K., Kurl S., Zaccardi F., Laukkanen J. A. (2016). Baseline and long-term fibrinogen levels and risk of sudden cardiac death: a new prospective study and meta-analysis. *Atherosclerosis*.

[20] Thompson S. G., Kienast J., Pyke S. D., Haverkate F., van de Loo J. C. (1995). Hemostatic factors and the risk of myocardial infarction or sudden death in patients with angina pectoris. European Concerted Action on Thrombosis and Disabilities Angina Pectoris Study Group. *The New England Journal of Medicine*.

[21] Howard S. C., Algra A., Rothwell P. M. (2008). Effect of age and glycaemic control on the association between fibrinogen and risk of acute coronary events after transient ischaemic attack or stroke. *Cerebrovascular Diseases*.

[22] Lip G. Y. H., Lowe G. D. O. (1995). Fibrin D-dimer: a useful clinical marker of thrombogenesis?. *Clinical Science*.

[23] Yarnell J. W., Baker I. A., Sweetnam P. M. (1991). Fibrinogen, viscosity, and white blood cell count are major risk factors for ischemic heart disease—the caerphilly and speedwell collaborative heart disease studies. *Circulation*.

[24] Thaulow E., Erikssen J., Sandvik L., Stormorken H., Cohn P. F. (1991). Blood platelet count and function are related to total and cardiovascular death in apparently healthy men. *Circulation*.

[25] Papageorgiou N., Tousoulis D., Siasos G., Stefanadis C. (2010). Is fibrinogen a marker of inflammation in coronary artery disease?. *Hellenic Journal of Cardiology*.

[26] Kotbi S., Mjabber A., Chadli A. (2016). Correlation between the plasma fibrinogen concentration and coronary heart disease severity in Moroccan patients with type 2 diabetes. Prospective study. *Annales d'Endocrinologie*.

[27] Yao H. Q., Wang F. J., Kang Z. (2016). Effects of endovascular interventions on vWF and Fb levels in type 2 diabetic patients with peripheral artery disease. *Annals of Vascular Surgery*.

